# Impact Behaviour of Steel-Fibre-Reinforced Alkali-Activated Slag Concrete Exposed to Elevated Temperatures

**DOI:** 10.3390/ma16114096

**Published:** 2023-05-31

**Authors:** Ahmed Abubakr, Ahmed Soliman

**Affiliations:** Gina Cody School of Engineering and Computer Science, BCEE, Concordia University, Montreal, QC H3G 1M8, Canada

**Keywords:** impact, A.A.S.C., residual properties, U.S.P.V., steel fibre, mechanical properties

## Abstract

Concrete protective structures are mainly meant to withstand impact loads. However, fire events weaken concrete and reduce its impact resistance. This study investigated the impact behaviour of steel-fibre-reinforced alkali-activated slag (AAS) concrete before and after exposure to elevated temperatures (i.e., 200 °C, 400 °C, and 600 °C). Hydration products’ stability under elevated temperatures, their effects on the fibre–matrix bond, and, consequently, AAS’s static and dynamic responses were investigated. The results reveal that adopting the performance-based design concept to achieve a balance between AAS mixtures’ performance under ambient and elevated temperatures is a crucial designing aspect. Advancing hydration products’ formation will increase the fibre–matrix bond at ambient temperature while negatively affecting it at elevated temperatures. High amounts of formed and, eventually, decomposed hydration products at elevated temperatures reduced the residual strength due to lowering the fibre–matrix bond and developing internal micro-cracks. Steel fibre’s role in reinforcing the hydrostatic core formed during impact loads and delaying crack initiation was emphasized. These findings highlight the need to integrate material and structure design to achieve optimum performance and that low-grade materials can be desired based on the targeted performance. A set of empirical equations for the correlation between steel fibre content in the AAS mixture and corresponding impact performance before and after fire exposure was provided and verified.

## 1. Introduction

Alkali-activated material (AAM) is a sustainable binding material incorporating precursors and alkali activators as raw materials. Many byproducts, such as fly ash, slag, and silica fume, are used as precursors to synthesize AAM [1]. Waterglass, potassium hydroxide, and sodium hydroxide are the most common activators used to activate these precursors [2]. AAM’s hydration products mainly depend on the precursor’s chemical composition and the activator properties [3]. In the case of (Ca + Si) precursors, such as slag, the formed hydration products are known to be mainly C-N-(A)-S-H and Hydrotalcite [4,5]. These hydration products have higher mechanical properties and stability than ordinary Portland cement (OPC) hydration products (i.e., mainly C-S-H and Portlandite). This can explain the higher mechanical properties and durability measures for AAM [6,7].

Moreover, the stability of AAM hydration products at elevated temperatures is higher than that of OPC systems [8,9]. For instance, Portlandite decomposes at elevated temperatures and rehydrates during cooling, exhibiting an increased volume [10]. This leads to internal stresses causing micro-crack development and mechanical property degradation. Hence, AAM systems have been suggested for use in critical structures that may be exposed to aggressive impact loads, such as railway buffers and machine padding [11]. Additionally, in protective structures, such as shelters and fortifications, concrete is supposed to suffer severe deterioration due to exposure to elevated temperatures induced by bombs or missiles. In such situations, concrete material is required to have a high absorbing capacity for impact energy induced by such high-strain-rate loadings and high stability during exposure to elevated temperatures.

Previous studies investigated the effect of strain rates on the behaviour of AAM, in which AAM showed lower strains at relatively higher stress levels regarding the applied loading strain rate [12]. This improvement in the performance compared to the OPC systems was attributed to the quality of the formed hydration products in the AAM binding systems.

Moreover, activator properties, such as silica modulus and dosage, were found to affect energy absorption under high-strain-rate loadings (i.e., impact). This was ascribed to the activator’s role in controlling the amount of formed hydration products and, consequently, microstructure development [13,14]. Activator dosage controls the precursor dissolution rate. The higher the dosage, the higher the dissolute spices from the precursor. Additionally, silica modulus dominates the supply of silica within the aqueous phase, contributing to the formation of three-dimensional hydration products due to the Si bridges’ high availability. This will eventually result in denser hydration products with high mechanical properties [1].

Many approaches, such as rubber incorporation, have been proposed to enhance concrete’s capacity to absorb impact energy [15,16,17]. However, increasing rubber’s incorporation level negatively affected the mechanical properties (i.e., compressive and tensile strength) [18,19,20]. Other studies have investigated the effect of incorporating different natural fibres, such as hemp and flax, or synthetic fibres, such as steel, basalt, and polypropylene fibres, in concrete [21,22,23]. Increasing the incorporation level of fibres improved concrete’s static and dynamic mechanical properties.

On the other hand, the effect of elevated temperatures on alkali-activated fibre-reinforced concrete is not well understood yet. Limited studies in the literature have investigated the behaviour of fibre-reinforced alkali-activated material under aggressive conditions, such as elevated temperatures. Generally, it was reported that alkali-activated slag mixtures exposed to elevated temperatures (i.e., around 600 °C) would retain almost 50% of their initial strengths compared to OPC mixtures, which lost their full strength [24]. As expected, fibre incorporation improved the mechanical properties of AAM at ambient temperature and after exposure to elevated temperatures [25,26]. Fibre addition to the matrix mitigates cracking due to thermal loadings induced by fire, resulting in better fire resistance. For instance, adding glass fibre could maintain 50% of AAS mortar strength at 500 °C, while with other types of fibres, similar behaviour can be achieved at 600 °C. Additionally, non-metallic fibre usually exhibits a higher strength loss [26]. The fibre type, content, and temperature level mainly control the improvements. Generally, elevated temperatures cause aggressive deterioration to the bond between the fibre and binder matrix and affect the fibre itself (i.e., based on its type), affecting the mechanical performance [27]. Moreover, a mixture reinforced by steel fibre will exhibit the maximum residual strength [26].

Therefore, in this study, the impact behaviour and the mechanical properties of plain and fibre-reinforced AAS concrete are evaluated at ambient temperature and after exposure to three levels of elevated temperatures (200 °C, 400 °C, and 600 °C). This will provide a better understanding of fibre incorporation rules in mitigating associated damages with exposure to elevated temperatures under impact loads for AAS.

### Research Significance

Extensive research in the literature has explored the effect of elevated temperatures on either mechanical or impact performance or both for AAMs without and with various fibre types. However, to the best of the authors’ knowledge, very limited studies have explored the impact behaviour of steel-fibre-reinforced alkali-activated slag concrete before and after exposure to elevated temperatures. Moreover, the existence of a positive relationship between mechanical and impact properties either at ambient or elevated temperatures was usually anticipated. Hence, most of the research has focused on maximizing the strength. However, this study highlights that this is not always true, and adopting a performance-based design concept while designing the mixture is more appropriate. Furthermore, this study highlights the compensating role of steel fibre addition for AAS microstructure degradation due to exposure to elevated temperatures. The ability of steel fibre to reinforce the hydrostatic core and delay crack initiation under impact loads is emphasized. A set of equations for correlations between steel fibre content and impact performance is established and validated.

## 2. Experimental Work

### 2.1. Materials

Granulated blast furnace slag (GBFS) with specific gravity and surface area of 2.92 and 515 m^2^/kg was used as the precursor for alkali-activated concrete. Table 1 summarises the chemical composition of the used slag. Sodium hydroxide and sodium silicate were used to prepare the activator solution. According to the manufacturer datasheet, sodium silicate contains 10.02% Na_2_O, 28.78% SiO_2_, and 62.2% H_2_O by mass. A high level of dosage and silica modulus activation was used to secure the good quality of the formed hydration product by increasing the dissolution rate and enriching the aqueous phase with silica spices. Crushed stones with a maximum nominal size of 19 mm were used as a coarse aggregate, with specific gravity and water absorption of 2.70 and 1.3% according to ASTM C33 [28], respectively. For the fine aggregate, natural riverside sand of specific gravity, fineness modulus, and water absorption of 2.70, 2.51, and 2.73%, respectively, were used for all concrete mixtures according to ASTM C136 [29] and ASTM C 128 [30]. For fibre-reinforced concrete mixtures, steel fibre with a specific gravity of 7.85, tensile strength of 1150 MPa, modulus of elasticity of 212 GPa, a diameter of 0.2 ± 0.02 mm, and a length of 14 ± 1 mm was added at rates of 0.25%, 0.5%, and 1% by volume.

### 2.2. Specimens’ Preparation and Exposure Regime

All concrete mixtures had a binder content of 400 kg/m^3^ and water-to-binder ratio of 0.44. The existing water in the sodium silicate was subtracted from the total water. Then, the additional water was used to dissolve the assigned amount of sodium hydroxide to achieve the targeted silica modulus (Ms) and the Na_2_O% dosage of 1.5 and 8%, respectively. Sodium hydroxide solution was prepared one day before concrete mixing and then left to cool down. All mixtures were prepared at a fine-to-coarse aggregate ratio of 1:1.75. Table 2 illustrates the compositions for all tested concrete mixtures. Concrete mixtures were prepared and cast following ASTM C192 [31]. Initially, dry sand, crushed stone, and slag were mixed for 1 min. Then, steel fibre was added and mixed for an extra 1 min to ensure uniform fibre distribution through the mixture. Then, the activator solution was added to the dry mixture and mixed for three additional minutes. Fresh density for all tested AAS concrete was evaluated according to ASTM C138.

All specimens were demoulded after 24 h, then stored in sealed bags after demoulding at the laboratory ambient condition (temp. 23 ± 1 °C and 45 ± 3% relative humidity). All specimens were exposed to elevated temperatures after 28 days to ensure the full formation of the hydration products. For each mixture name, the letters (PC or SFC) refer to plain concrete or steel-fibre-reinforced concrete, followed by the steel content ratio by volume, respectively.

Cylindrical concrete specimens of a diameter of 100 mm and length of 200 mm were prepared and tested according to ASTM C39 [32], and ASTM C496 [33], respectively. For the modulus of elasticity, specimens’ deformation at the middle portion was monitored using a compressometer with two linear variable differential transducers connected to a data acquisition system. The specimens were loaded to 40% of their compressive strength. The slope of the developed stress–strain curve was used to calculate the modulus of elasticity according to ASTM C469 [34]. Specimens were tested at ambient temperature at ages 7 and 28 days. All reported results represent the average of at least two samples. The maximum coefficients of variation for compressive strength, tensile strength, and modulus of elasticity results were 7.4%, 9.3%, and 5.2%, respectively. A drying shrinkage test was performed on prismatic specimens 75 × 75 × 285 mm from each mixture according to ASTM C 157 [35]. The unrestrained one-dimensional deformations were measured using a dial gauge with an accuracy of 10 μm/m. Shrinkage prisms were measured daily for the first week after casting, then weekly for up to 56 days. Thermogravimetric analyses for selected binder samples were performed. Samples weighed around 30 mg from ground binder sieved on sieve no. 200 and were heated at a rate of 5 °C/min till 650 °C. For the ultrasonic pulse velocity test (USPV), 75 × 75 × 285 mm prismatic specimens were prepared. The direct transmission method was employed according to ASTM C597 [36]. Initially, the testing specimen was placed on a levelled and stable surface. The specimen’s length along the direction of the ultrasonic waves was measured. After applying a coupling agent, two transducers were coupled to the specimen’s surfaces. This polymeric gel ensured full contact between the transducers and the specimen’s surfaces. Moreover, the two transducers were aligned on opposite surfaces, so the measured dimensions and assumed opposite travel paths were identical. An average of three measurements per spot on the specimen surface was reported. Moreover, the dynamic modulus of elasticity (E_d_) was calculated for all mixtures as a function of the bulk density and the ultrasonic pulse velocity for different mixtures using the following equation (Equation (1)):

Ε_d_ = (V^2^*ρ*/*g*) (1)

where E_d_ is the dynamic modulus of elasticity in GPa, V is the ultrasonic pulse velocity in km/s, *ρ* is the concrete density in kg/m^3^, and *g* is the gravity acceleration in cm/s^2^.

**Figure 1:**
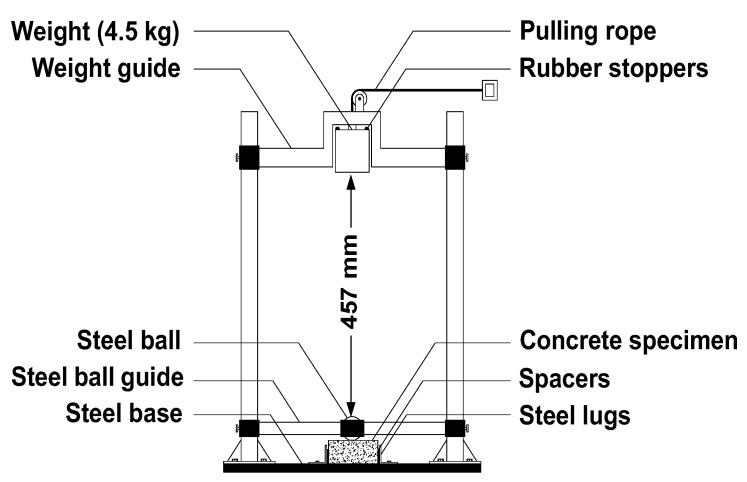
Drop weight impact machine.

For the impact test, 150 mm diameter cylindrical samples with 63 mm height were cast to be tested according to ACI. Committee 544 (drop weight test). In this test, a steel ball of 63.5 mm diameter is placed on the top of the concrete sample; then, a 4.5 kg weight is dropped on this steel ball from a height of 457 mm. Then, the number of successive blows until failure is counted. The total absorbed energy by a concrete sample was equivalent to the total number of blows multiplied by the energy amount transferred to the sample per blow. Figure 1 indicates the impact test machine used for the drop weight impact test.

**Figure 2:**
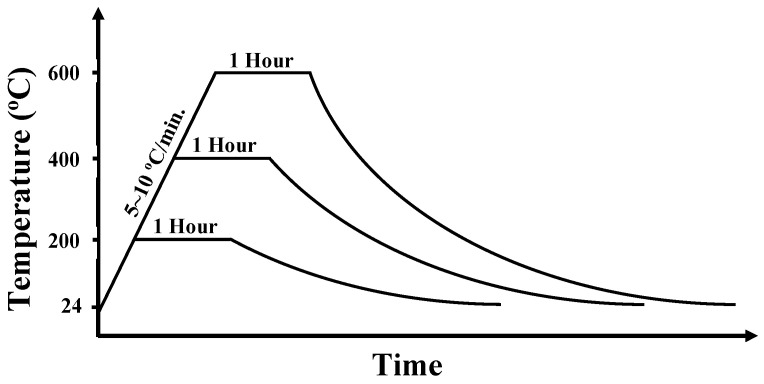
The regimes of elevated temperature exposure.

The used regime for elevated temperature exposure at testing age is briefed in Figure 2. Samples were heated to 200 °C, 400 °C, and 600 °C. The 5–10 °C/min heating rate was fixed for all targeted temperatures according to ASTM E831 [37]. Then, specimens were kept exposed to the assigned temperature for one hour. Afterwards, specimens were left to cool down in the oven.

Moreover, to ensure that the specimen core reached the targeted temperature, the temperature at the centre of a dummy specimen was monitored using a thermocouple type K. Specimen’s temperature, along with the oven temperature, was continuously monitored and recorded using a data acquisition system.

## 3. Results and Discussion

Generally, increasing the fibre content increased the concrete density due to the relatively high density of steel fibre. For instance, plain AAS concrete showed a density of 2448.5 kg/m^3^, compared to 2502 kg/m^3^ for mixtures incorporating 1% steel fibre. As expected, adding fibre significantly reduced the workability of concrete [38]. However, several studies confirmed that mixtures incorporating steel fibre, with volume fractions up to 1.0%, can still achieve the desired workability [39]. Moreover, using vibration during placement facilitated the casting and offset the reduction in the workability [40].

### 3.1. Compressive Strength

**Figure 3:**
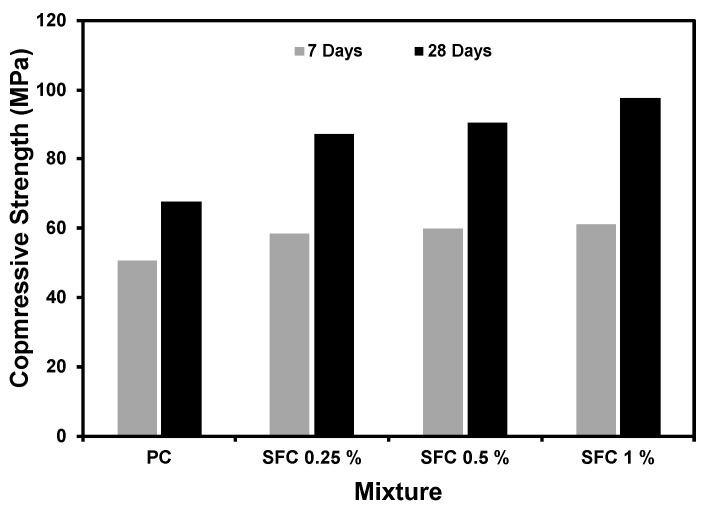
Compressive strength of plain and steel fibre concrete at ambient temperature.

Compressive strength results for all mixtures at 7 and 28 days are presented in Figure 3. Steel fibre was very beneficial to the AAS concrete in terms of strength, regardless of its addition rate agreeing with previous findings [41,42,43,44]. It enhanced the concrete behaviour at pre- and post-cracking zones [45]. Before cracking, steel fibre enhanced the concrete ductility, leading to a higher load-carrying capacity during the elastic zone [46]. This can be attributed to the excellent bond between the fibre and the surrounding AAS binder. Hence, the load sufficiently transferred from the binder to the steel fibre, leading to a successful unity between the binder and fibre to behave harmonically [47]. Moreover, after the elastic zone, steel fibre will increase the concrete capacity to absorb more straining energy through bridging cracks, leading to a higher compression load peak and toughness before failure [48]. This agrees with the modulus of elasticity results, as illustrated later. Moreover, the higher the fibre content, the greater the achieved ultimate compressive strength at age 28 days. This reflected the optimized benefits of enhancing the pre- and post-cracking of concrete behaviour within the acceptable workability limits of fresh concrete. For instance, specimens incorporated with 1% steel fibre by volume achieved a 44% increase in compressive strength than that of plain mixtures at 28 days.

**Figure 4:**
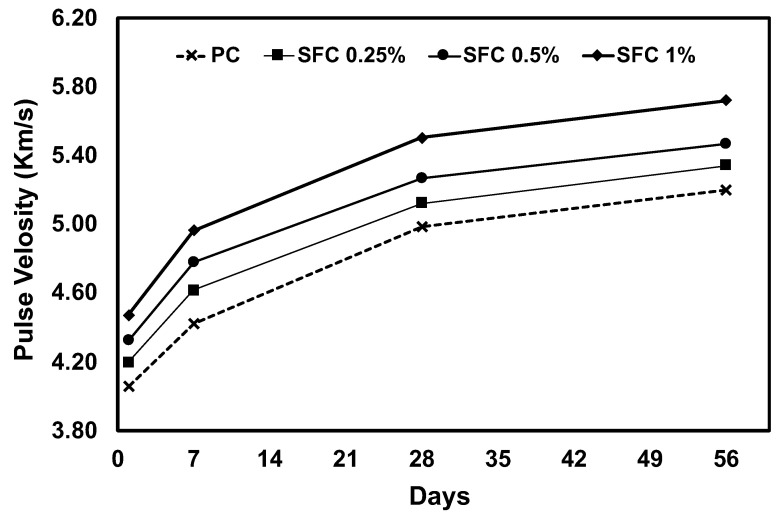
USPV of all concrete mixtures.

These compressive strength results were confirmed with USPV measurements for AAS concrete specimens at different ages. The ultrasonic pulse’s measured velocities through the concrete matrix increased as fibre content increased (Figure 4). This can be attributed to the higher density of the steel, resulting in a higher pulse velocity (i.e., −1.2 the velocity through concrete) [49]. This increases the chance of the signal passing through the fibre with higher velocity instead of turning around, increasing the elapsed time before pulse receiving due to a longer path. The higher density of steel fibre can explain the increased compressive strength along with its bridging action to hold the crack propagation due to compression loads. Moreover, USPV increased for all mixtures with time. This can be ascribed to the decrease in void due to further hydration and formation of hydration products leading to an increase in the gel/space ratio.

**Figure 5:**
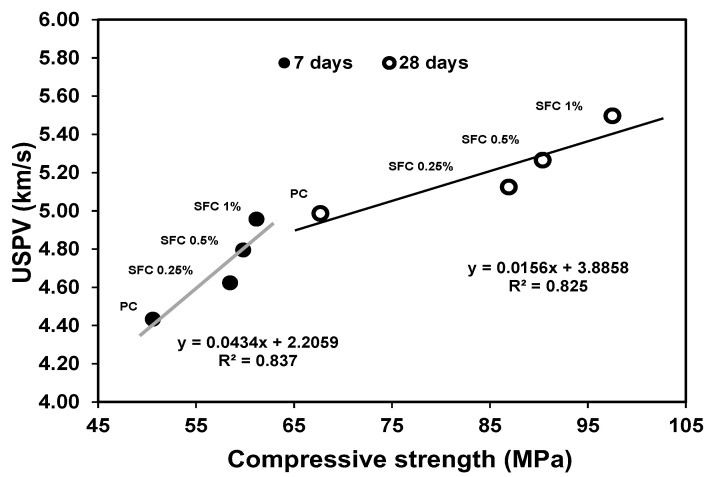
Relationship between compressive strength and USPV for different fibre content at different ages.

Figure 5 shows the relationship between the measured USPV and the achieved compressive strength for different fibre contents. The USPV increased as the specimen density increased by the high-density fibre addition with higher mechanical properties and, consequently, high load-carrying function during the compression loading, leading to higher compressive strength in an exponential relationship with regression >0.8. This indicates the strong correlation between the compressive strength and the specimens’ USPV measures.

**Figure 6:**
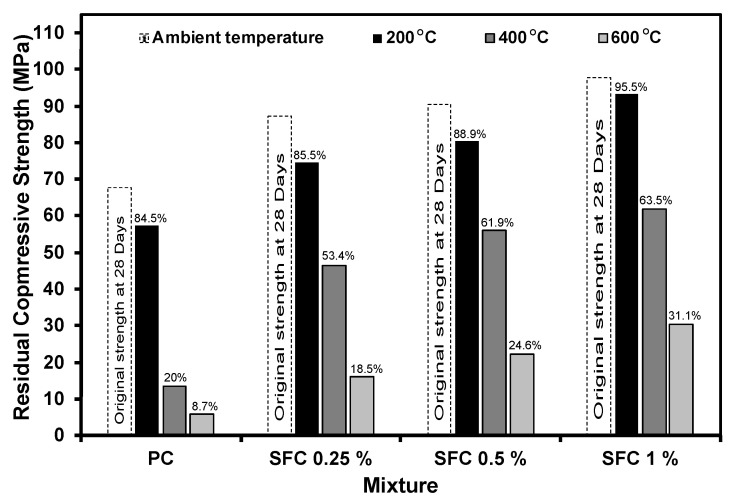
Residual compressive strength of all concrete mixtures after exposure to different levels of elevated temperatures.

Figure 6 illustrates the residual strengths of SFC and PC after exposure to various elevated temperature levels. As expected, SFC mixtures exhibited higher residual strengths than PC mixtures as fibre addition offset the negative effect of binder deterioration induced by exposure to elevated temperatures. The higher the fibre content, the greater the residual compressive strength. It should be mentioned that the PC mixture suffered significant strength reductions as exposure temperatures exceeded 200 °C. For instance, PC exhibited a residual strength of 85% of the original strength after exposure to 200 °C, while it achieved only 23% of the original strength after exposure to 400 °C. This is ascribed to the severe micro-cracking development due to the build-up of pore pressure induced at relatively high levels of elevated temperatures [8,50]. This will also affect all the concrete mechanical properties along with the impact absorption capacity, as explained later. However, steel fibre incorporation could mitigate this effect, especially at high fibre contents.

Similar to PC mixtures, SFC mixtures exhibited a reduction in residual strengths as exposure temperature increased. However, all SFC residual strengths were higher than the PC mixtures. Moreover, at higher exposure temperatures (above 200 °C), the enhancements in the residual strength for SFC mixtures compared to PC mixtures were more quantified. For instance, the mixture with 1% steel fibre exhibited 30.4 MPa compressive strength compared to 6 MPa for PC specimens after exposure to 600 °C. Hence, it is clear that steel fibre addition has a high potential to enhance the AAS compressive strength before and after exposure to elevated temperatures.

It should be mentioned that another factor contributing to the high residual strengths for AAS compared to the PC mixture is the absence of explosive spalling at the tested temperature range. This was mainly attributed to the change in the pore structure and its connectivity, which facilitates the release of the internal steam pressure, leading to lower tensile stresses and spalling risk [51,52,53,54,55,56].

### 3.2. Tensile Strength

**Figure 7:**
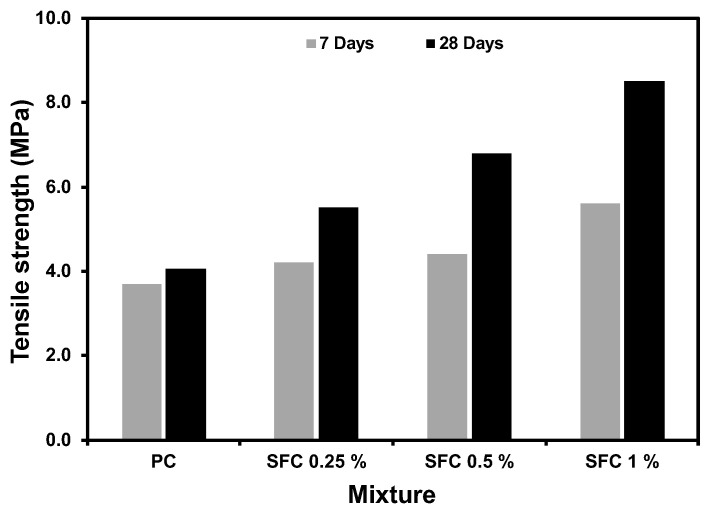
Tensile strength of plain and steel fibre concrete at ambient temperature.

**Figure 8:**
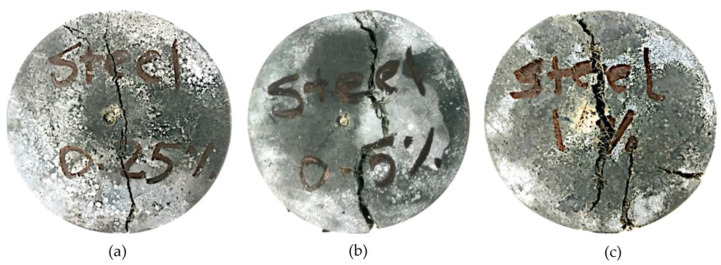
Specimens failure after tensile strength test at ambient temperature (**a**) specimen with 0.25% fibre, (**b**) specimen with 0.5% fibre and (**c**) specimen with 1% fibre).

Figure 7 summarises all concrete mixtures’ tensile strength results at 7 and 28 days at ambient temperatures. The enhancement in tensile strength due to steel fibre addition was significant and even higher than that of the compressive strength. For instance, mixtures with 1% steel fibre exhibited almost double the plain concrete mixture tensile strength, while the enhancement in compressive strength was 44%. This is consistent with previous research [57]. Fibre-bridging action minimizes crack development and delays crack development and propagation during tensile loading. Hence, higher tensile strength can be achieved. Visual inspection for tested specimens confirmed the crack bridging action as the specimen could hardly separate into two parts, as shown in Figure 8a–c.

**Figure 9:**
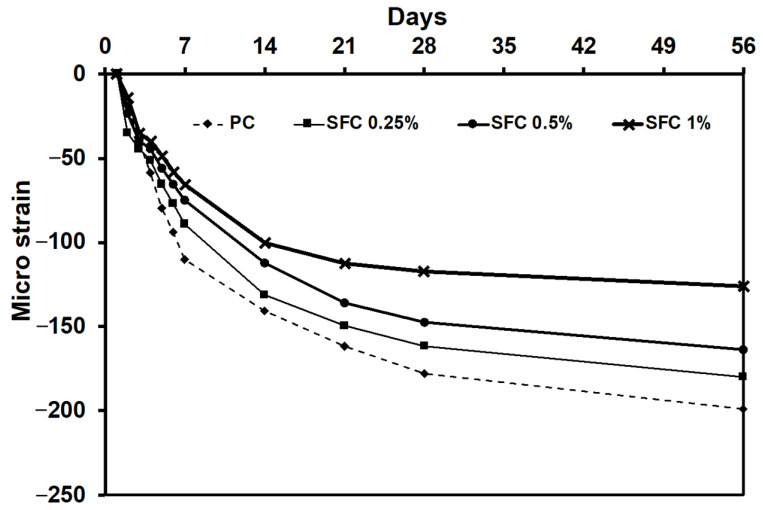
Concrete drying shrinkage micro-strain.

**Figure 10:**
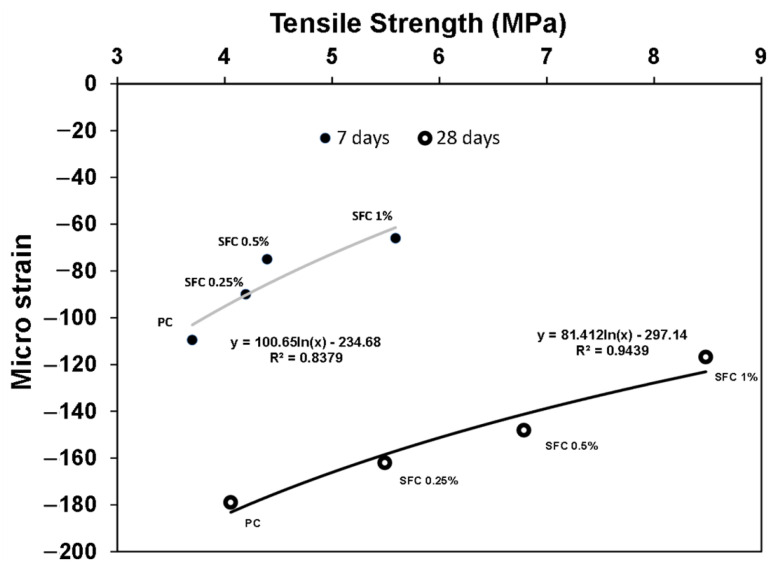
Correlation between tensile strength and developed drying shrinkage.

Moreover, the measured micro-strain drying shrinkage for SFC was lower than that of the PC. The higher the fibre content, the lower the micro-strain drying shrinkage (Figure 9). During the drying shrinkage, the coarse aggregates partially restrain crack development in the surrounding binder. However, the steel fibre compensated for this action, resulting in a lower cracking density, which reduced the pre-existed micro-cracks and increased the concrete tensile strength [58]. This was confirmed by directly correlating the tensile strength and the developed drying shrinkage micro-strain (Figure 10). Increasing the micro-strain can be assigned to the concrete tensile strength decreasing.

**Figure 11:**
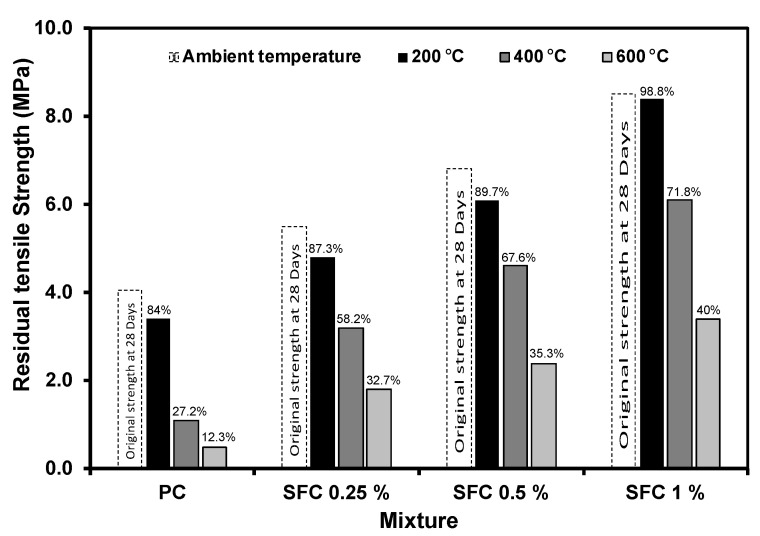
Concrete residual tensile strength after exposure to different levels of elevated temperatures.

After exposure to various elevated temperatures, PC tensile strength suffered a considerable loss, as shown in Figure 11. This can be attributed to binder degradation during exposure to elevated temperatures. Additionally, concrete brittleness increased, leading to a noticeable reduction in the tensile strength [59]. For instance, the tensile strength of PC after exposure to 400 °C was reduced by 73% compared to that at ambient temperature. However, steel fibre kept the specimen integrity during the tensile test, exhibiting a great enhancement in tensile strength compared to PC. Generally, high steel fibre content increases the residual tensile strength regardless of the elevated temperature level. For instance, after exposure to 600 °C, specimens with 1% of steel fibre showed around a reduction of only 6% in tensile strength than the PC at ambient temperature. Moreover, specimens with 1% of steel fibre tensile strength after exposure to the highest temperature (i.e., 600 °C) were better than that of PC exposed to the lowest elevated temperature (i.e., 200 °C).

### 3.3. Modulus of Elasticity and Modulus of Resilience

The elastic modulus of fibre-reinforced concrete relies on the modulus of elasticity and tensile strength for both the matrix and used fibre, along with the bond between them [60]. Increasing the steel fibre content at ambient temperature increased the concrete modulus of elasticity. This agrees with previous findings [44,46,61,62,63,64,65,66,67,68,69,70]. The relatively high modulus of elasticity for steel fibre and good bond with the surrounding matrix limited the strain development during loadings. For instance, as steel fibre content increased from 0.25% to 1%, the modulus of elasticity increased in the range of 13% up to 23% compared to the PC mixture. On the other hand, at elevated temperatures, the binder deterioration dominated the concrete’s elastic modulus changes as it reduced the matrix–fibre bond. Consequently, steel fibre’s ability to limit strain development during loadings will be lower in specimens exposed to elevated temperatures than in other specimens at ambient temperature. Moreover, increased binder brittleness due to elevated temperature exposure will lower the load capacity within the concrete elastic behaviour zone. Consequently, earlier cracks will develop and propagate due to the insufficient restraining induced by the used fibre through bridging these cracks.

**Figure 12:**
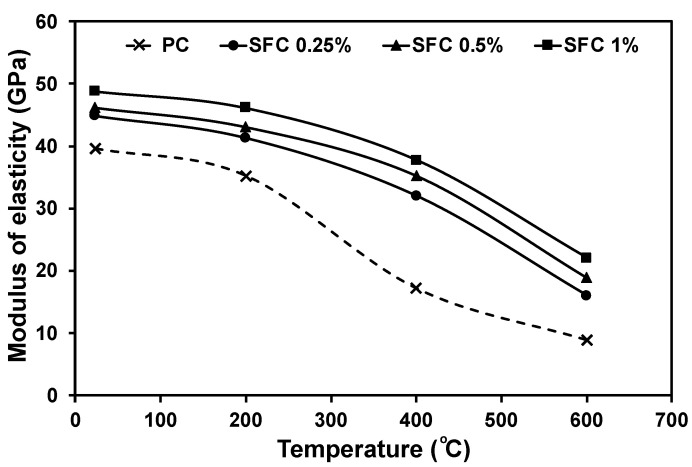
Concrete modulus of elasticity at ambient temperature and after exposure to various elevated temperatures.

Plain concrete mixtures suffered notable degradations in the modulus of elasticity at 400 °C and 600 °C by losing about 57% and 78% of their original value at ambient temperature, respectively. However, steel fibre addition resulted in a higher elastic modulus than PC. It showed a uniform reduction at various temperature levels (Figure 12). For instance, 1% steel fibre concrete achieved an elastic modulus of 47.17 GPa, 37.8 GPa, and 22.1 GPa, compared to 35.2 MPa, 17.2 MPa, and 8.9 MPa achieved by PC after exposure to 200 °C, 400 °C, and 600 °C, respectively.

**Figure 13:**
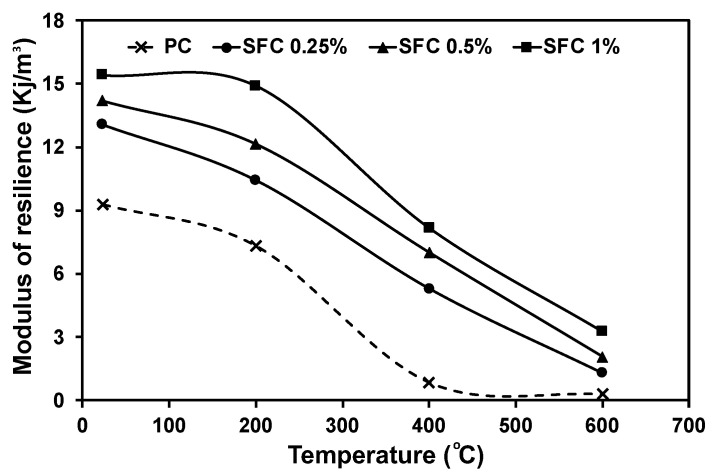
Concrete modulus of resilience at ambient temperature and after exposure to various elevated temperatures.

Additionally, the dynamic modulus of elasticity (Ed) at ambient temperature increased by increasing the steel fibre content in the mixture. For instance, Ed for SFC mixtures containing 0.5% steel fibre was 70.1 GPa compared to 62.1 GPa for PC. In addition, the modulus of resilience, which represents the concrete capability of retrieving the induced strain during loading within the elastic zone, was enhanced as the fibre content increased. This mainly depends on the concrete’s strain energy absorption capacity before cracking, limiting the retrieved strain after unloading. Figure 13 represents the resilience modulus achieved by PC and SFC with different steel contents at ambient temperature and after exposure to various elevated temperatures. Steel fibre addition significantly enhanced the concrete modulus of resilience at ambient temperature. However, after exposure to elevated temperatures, all mixtures’ resilience modulus exhibited a reduction trend as the exposure temperature increased. Fibre-reinforced concrete mixtures reserved a relatively higher concrete resilience than plain mixtures. For instance, the resilience moduli of concrete mixtures containing 1% steel fibre were 14.9 kJ/m^3^, 8.2 kJ/m^3^, and 3.3 kJ/m^3^ compared to 7.3 kJ/m^3^, 0.8 kJ/m^3^, and 0.3 kJ/m^3^ for PC specimens after exposure to 200 °C, 400 °C, and 600 °C, respectively. This indicates the great ability of steel fibre to enhance the concrete resilience modulus after exposure to elevated temperatures.

### 3.4. Impact Energy Absorption

**Figure 14:**
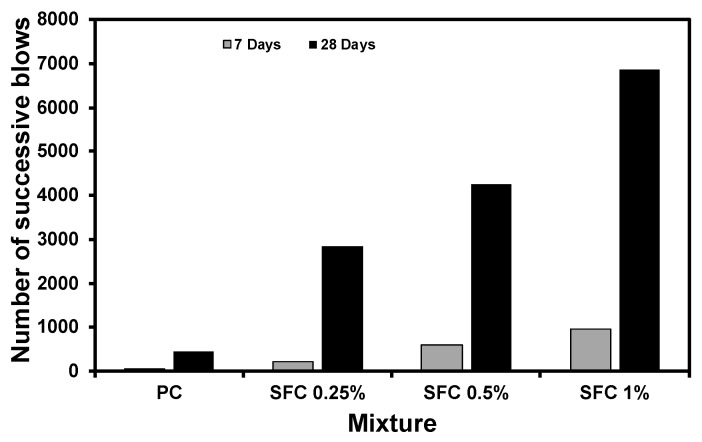
Concrete impact energy absorption capacity at ambient temperature.

Figure 14 illustrates the number of successive blows received by concrete mixtures before failure during the drop weight impact test. The number of successive blows reflects each mixture’s impact energy absorption capacity. The impact energy absorption capacity will be equivalent to the total accumulative impact energy delivered by these blows [71]. Generally, SFC mixtures exhibited higher impact energy absorption than PC mixtures at ambient temperature. PC mixtures absorb impact energy until the first crack development, representing the ultimate capacity. Conversely, in SFC mixtures, the fibre existence controlled the specimen strain during the impact event, consequently limiting crack initiation up to a higher level of absorbed impact energy than PC [45]. Moreover, as previously mentioned, the higher resilience achieved through fibre incorporation increased the specimen’s internal stress capacity above which cracks would be initiated.

**Figure 15:**
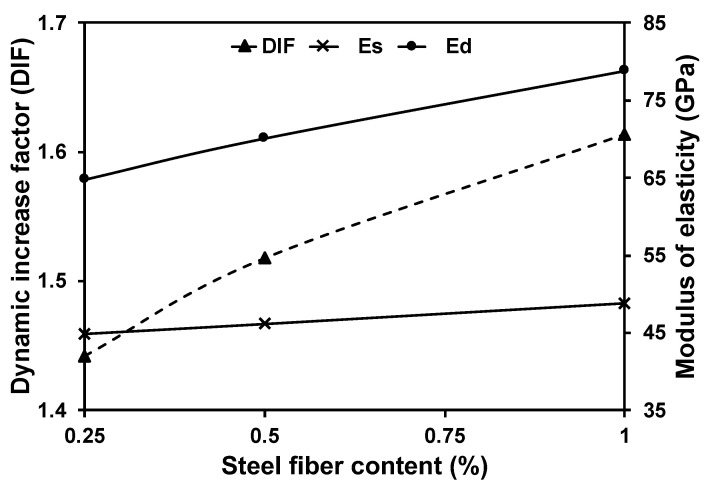
Steel fibre concrete dynamic increase factor (DIF).

Impact loads induce a higher strain rate than static loading [72]. Hence, concrete will possess higher resistance to impact loads. This can be quantified by measuring the dynamic increase factor (DIF) achieved by various concrete mixtures. The DIF is the magnification of the specimen’s stress capacity under dynamic loading relative to static loading. It was observed that the higher the steel fibre content, the higher the achieved DIF (Figure 15). This could explain the considerable enhancement in impact resistance. For instance, the PC specimen initially cracked after 442 blows, compared to 2366, 3678, and 6024 successive blows during the impact test for mixtures incorporating 0.25%, 0.5%, and 1% steel fibre at age 28 days.

**Figure 16:**
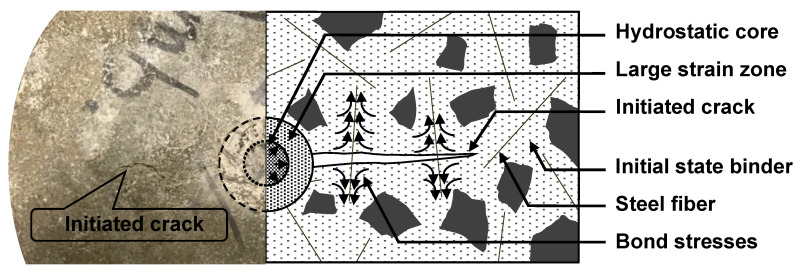
Steel fibre mechanism to limit the initiated crack propagation.

During the impact test and directly beneath the steel ball, a damaged mechanism started to occur. Due to considerable compression applied by the steel ball during the impact event on the contact surface with the specimen, a hydrostatic core started to form. This core started to collapse, transferring the pressure to the surrounding zone and inducing large strains. Once strains in this zone exceed the elastic limit of concrete, cracks start to initiate and propagate to the outer zone (i.e., the initial state zone). After cracking, the fibre and matrix–fibre bond will dominate the resistance behaviour towards higher impact loads (Figure 16). As mentioned earlier, increasing the steel fibre content was beneficial by bridging action and limiting crack propagation. This significantly increased the AAS concrete toughness after the first crack development, resulting in a higher number of successive blows until failure (i.e., the ultimate impact absorption capacity). For instance, mixtures incorporating 0.5% and 1% steel fibre needed 566 and 836 blows after the first visible crack to achieve the failure criteria crack compared to only 8 blows for the PC mixture.

**Figure 17:**
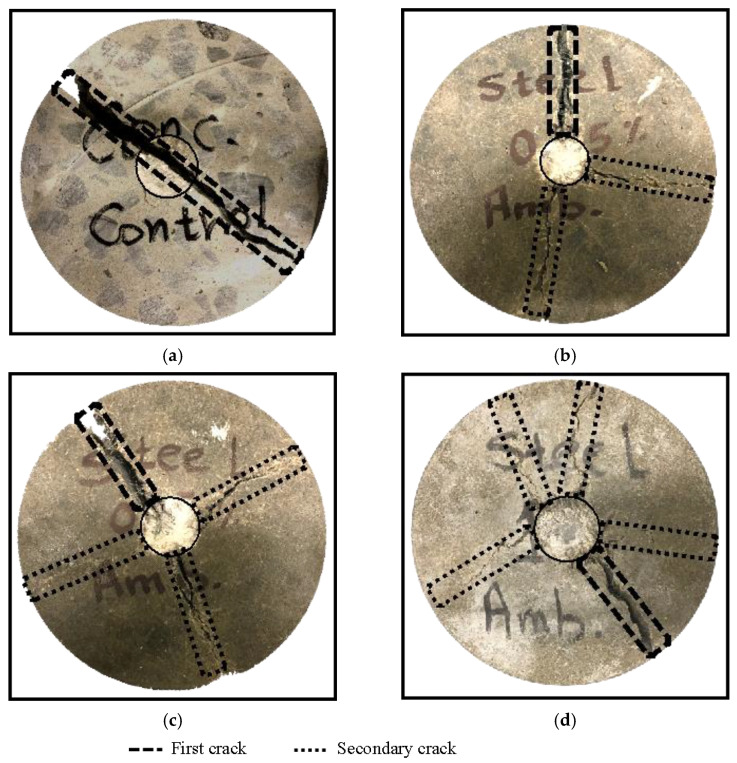
First and secondary cracks developed at the failure of AAS concrete (**a**) plain concrete, (**b**) 0.25% steel fibre, (**c**) 0.5% steel fibre, and (**d**) 1% steel fibre.

The number of secondary initiated cracks developed after the first visual crack and up to specimen failure confirmed the different mixtures’ toughness during the impact test. Secondary cracks are initiated based on steel fibre’s ability to contain and limit the first crack propagation before achieving the failure criteria [45]. For instance, the PC specimen had only one crack upon failure. However, increasing the steel fibre content increased the number of secondary cracks (Figure 17).

**Figure 18:**
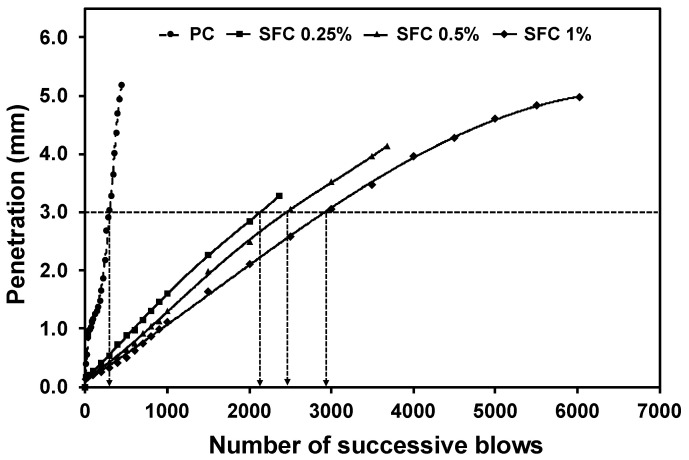
Penetration level by steel ball below AAS fibrous surface and the plain concrete surface (Dotted line shows the 3 mm penetration level).

In another case, the steel ball’s penetration level under the specimen’s surface was monitored during the impact test. The achieved penetration level reduced as the steel fibre content increased. This can be attributed to the steel fibre existence, especially in the large strain zone. In this zone, steel fibre limits the strains to maintain the AAS concrete’s elastic limit up to higher stress levels than the PC mixtures. As a result, the crushing zone (i.e., the hydrostatic core previously) will be limited, leading to a lower steel ball penetration level. For instance, a penetration level of 3 mm was achieved after 2460 successive blows for mixtures incorporating 0.50% steel fibre compared to 300 blows for the PC specimen (Figure 18). This showed the significant effect of the steel fibre addition in resisting ball penetration during the impact event.

**Figure 19:**
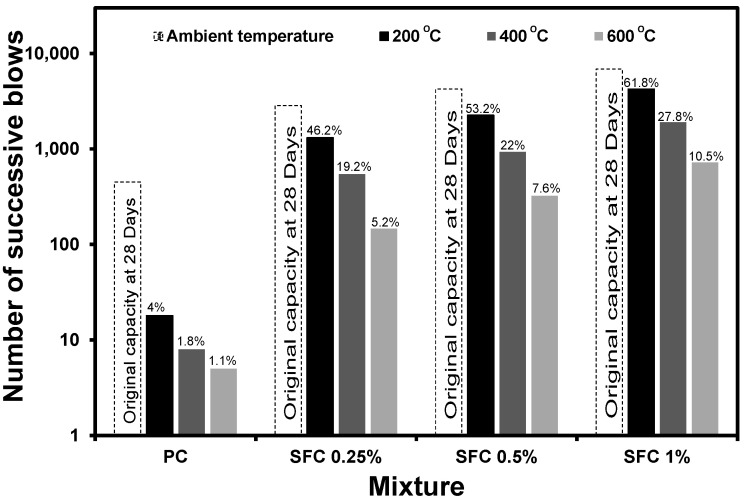
Residual impact absorption capacity of SFC after exposure to various elevated temperatures.

After exposure to various elevated temperatures, all SFC mixtures achieved significantly higher impact absorption capacity than PC mixtures, regardless of the fibre content. For instance, after exposure to 600 °C, SFC mixtures containing 1% steel fibre absorbed the accumulative impact energy induced by 720 successive blows, compared to 5 blows only for PC exposed (Figure 19).

This can show the quantum leap achieved in the impact behaviour of AAS concrete after exposure to various elevated temperatures by adding steel fibre. Steel fibre significantly limited the crack development in concrete, which started by reducing drying shrinkage micro-cracking at early stages (Figure 9). It also minimized the effect of pore water pressure due to exposure to elevated temperatures, achieving the minimum level of induced micro-cracking [73]. Finally, it showed excellent behaviour during the impact event. SFC mixtures achieved a significantly enhanced resilience, which upgraded the concrete strain capacity level to a considerably higher impact energy level. Moreover, the high penetration resistance achieved by the steel fibre inclusion delayed the crack initiation from the hydrostatic core to the initial state of concrete.

**Figure 20:**
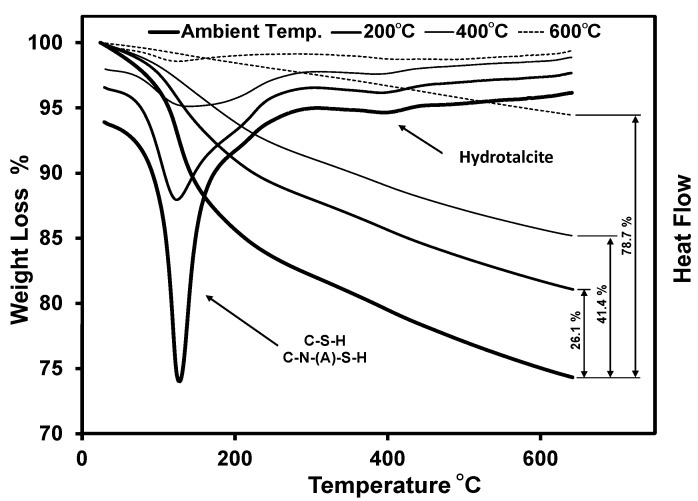
Thermogravimetric analysis of the binder before and after exposure to various elevated temperatures.

Besides the steel fibre content, the binder deterioration will affect the impact behaviour after exposure to elevated temperatures. As the binder is exposed to elevated temperatures, different hydration products start to decompose according to each product’s stability. This decomposition will lead to binder weight losses and a reduction in density [74]. This will eventually result in a lower matrix–fibre bond. The weight loss and the heat flow of the tested sample before and after exposure to each elevated temperature were compared to assess the binder quality after exposure to elevated temperatures. It was found that increasing the temperature exposure level before the TGA test resulted in a higher relative weight loss and, consequently, lower binder density compared to that of the sample tested without exposure to an elevated temperature. For instance, tested samples after exposure to 400 °C exhibited 41.4% relative weight loss with respect to that of the sample without exposure to elevated temperature prior to testing (Figure 20). This illustrated the effect of each elevated temperature individually on the binder.

**Figure 21:**
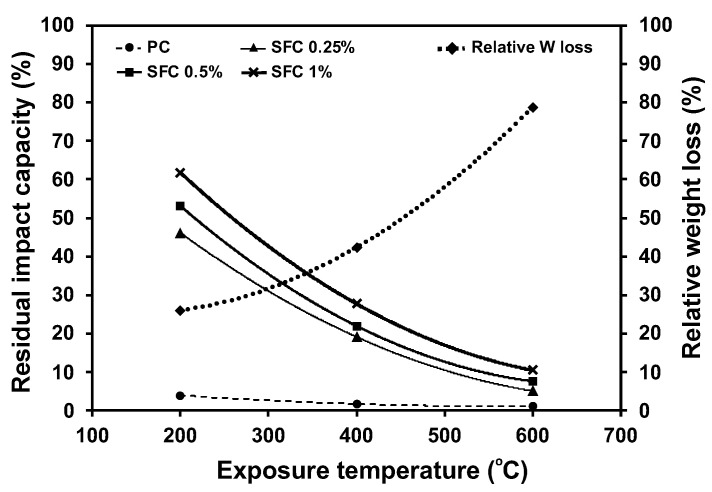
Residual impact energy absorption vs. the binder weight losses after exposure to elevated temperatures.

On the other hand, steel fibre incorporation efficiently compensated for the negative effect of elevated temperatures on the binder. Figure 21 shows the relative residual impact absorption capacity for each mixture, which is the impact absorption capacity after exposure to elevated temperatures with respect to its original capacity at ambient temperature. In the same figure, the relative weight loss of the binder after exposure to elevated temperature was illustrated with respect to the weight loss without exposure to elevated temperature during the TGA test. It can be observed that while the deterioration of the binder has a fixed effect on the specimens’ impact absorption capacity, the relative residual impact absorption increased at each temperature level as fibre content increased. For example, mixtures containing 0.25%, 0.5%, and 1% steel fibre exhibited 46%, 53%, and 62% residual impact strength compared to the 4% residual impact absorption capacity achieved by the PC mixture. This indicates the great enhancement in the impact absorption after exposure to elevated temperatures achieved by increasing the steel fibre content.

Moreover, the SFC mixtures’ toughness was considerably higher than that of the PC mixture after exposure to elevated temperatures. Table 3 represents the number of blows up to the first crack (Nc) along with the failure number of blows (Nf) for mixtures with different steel fibre contents after exposure to different temperatures.

**Figure 22:**
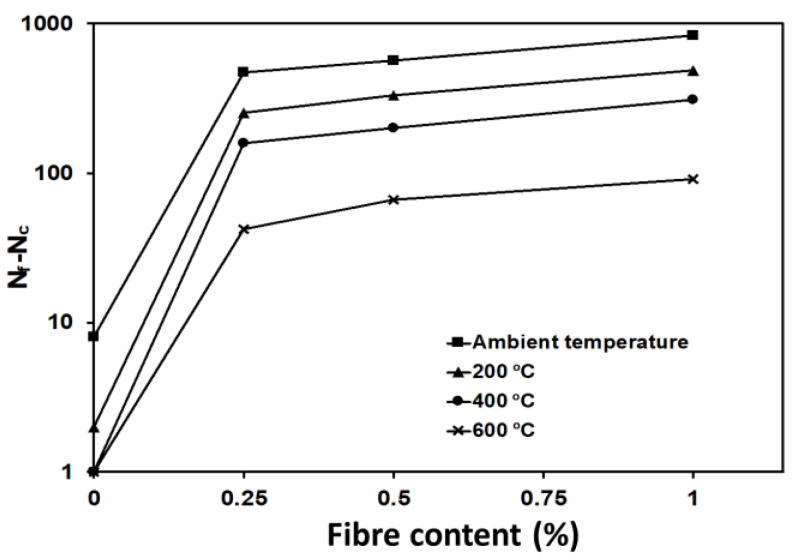
Number of successive blows after the first visual crack.

The number of blows required to achieve the failure criteria after the development of the first visual crack was found to increase by increasing the steel fibre content at all elevated temperature levels. For example, after exposure to 200 °C, specimens containing 0.25%, 0.5%, and 1% of steel fibre required more than 256, 334, and 492 blows to fail compared to only 2 blows needed by plain concrete (Figure 22). This can be ascribed to the reserved capability of steel fibre to limit the crack propagation in the decomposed binder, maintaining high toughness before failure under impact loads.

### 3.5. Relationship between Fibre Content and Impact Absorption Capacity

**Figure 23:**
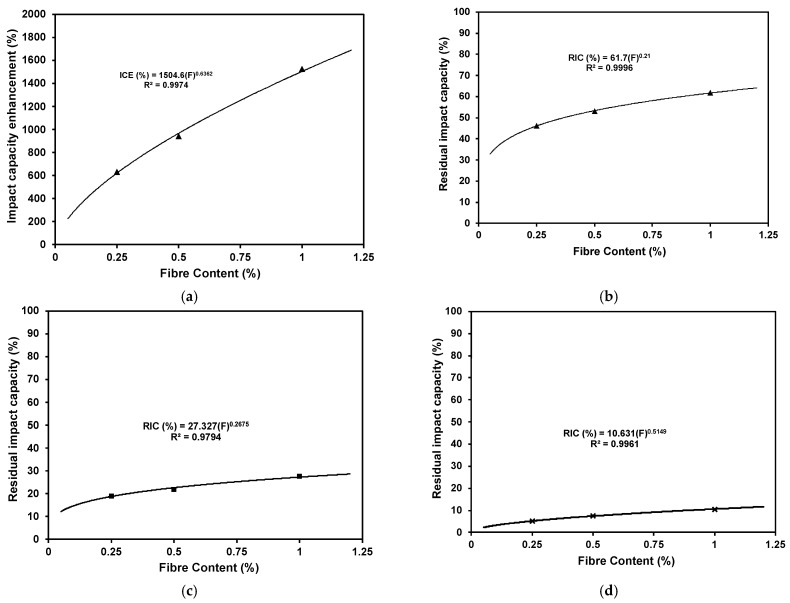
Relationship between fibre content and the enhancement percentage in impact absorption capacity for specimens (**a**) at ambient temperature, (**b**) after exposure to 200 °C, (**c**) after exposure to 400 °C, and (**d**) after exposure to 600 °C.

Figure 23a–d plot the relationship between the fibre content (%) as a volume fraction and the achieved impact capacity enhancement (ICE) at ambient temperature and the residual impact capacity (RIC) after exposure to various elevated temperatures. The results illustrate a direct correlation between the steel fibre content and the increased impact energy absorption before and after exposure to elevated temperatures. The best relationship representing the increasing trend was a power equation. R2 values of 0.997, 0.999, 0.979, and 0.996 at ambient temperature and after exposure to 200 °C, 400 °C, and 600 °C, respectively, confirm that the following equations can yield a very accurate estimation of the enhancement achieved by fibre incorporation.

ICE (Ambient) = 1504.6 (F)^0.6362^ (2)

RIC (200 °C) = 61.7 (F)^0.21^ (3)

RIC (400 °C) = 27.327 (F)^0.2675^ (4)

RIC (600 °C) = 10.631 (F)^0.5149^ (5)

where ICE is the impact capacity enhancement (%) at ambient temperature, RIC is the residual impact capacity after exposure to elevated temperatures, and F is the fibre content (%) as a volume fraction.

## 4. Conclusions

In this study, the static (i.e., mechanical) and dynamic (i.e., impact) properties of steel-fibre-reinforced AAS mixtures before and after exposure to various levels of elevated temperatures were evaluated. Moreover, the correlations between steel fibre contents and various properties were also illustrated and supported with empirical equations. The following points summarise the main findings of the study:

1. The steel fibre incorporation greatly enhanced the mechanical and impact properties for the AAS concrete before and after exposure to elevated temperatures, which can be attributed to its efficiency in bridging micro-cracks, increasing ductility, and restraining deformation.
2. At ambient temperature, AAS concrete impact behaviour was efficiently enhanced by steel fibre incorporation due to the increased concrete resilience. The higher the steel fibre content, the higher the impact energy absorption capacity.
3. Steel fibre addition had a higher positive impact on AAS concrete properties at elevated temperatures as its restraining effect compensates for the degradation of AAS concrete due to the decomposition of hydration products.
4. During the impact loading, steel fibrous AAS concrete had higher penetration resistance than plain concrete as the steel fibre reinforced the hydrostatic core zone formed during impact, thereby delaying crack initiations.
5. A direct correlation between the steel fibre content and the achieved enhancement in the impact absorption capacity before and after exposure to various elevated temperatures was proposed.

## 5. Recommendation for Future Work

1. There is a current trend to utilize various powder activators to generate one-part alkali-activated materials. Hence, the effects of changing the nature of the activator and its stability at elevated temperatures on the impact behaviour need more investigation.
2. Only one type of steel fibre was evaluated. However, there is potential to have variations in the results as the shape and size of the steel fibre are changed.
3. We suggest evaluating the mechanical and impact properties for hybrid-fibre-reinforced AAS to optimize the mixture to achieve optimum performance.
4. Several techniques for enhancing both the mechanical and impact performance of concrete exist (such as the addition of rubber). Hence, combining various techniques with fibre could lead to better performance.
5. Further research is needed to develop a predictive model.

## Figures and Tables

**Table 1:** Chemical composition of slag.

Constituent/Property (%)	Slag
SiO_2_	36.1
Al_2_O_3_	10.1
CaO	37
MgO	12
K_2_O	0.5
Na_2_O	0.4
Fe_2_O_3_	0.7
SO_3_	3
Other Minor Elements	0.2

**Table 2:** Concrete mixture proportions.

Mixture	Slag (kg/m^3^)	Fine agg. (kg/m^3^)	Coarse agg. (kg/m^3^)	Steel Fibre by Volume (%)	Steel Fibre (kg/m^3^)	NaOH (kg/m^3^)	Na_2_SiO_3_ (kg/m^3^)	Water (L/m^3^)
PC	400	650	1137.5	0	0	21.9	166.8	72.2
SFC 0.25%	399	648.4	1134.7	0.25	19.625	21.85	166.4	72.1
SFC 0.5%	398	646.8	1131.8	0.50	39.25	21.8	165.9	71.9
SFC 1%	396	643.5	1126.2	1.00	78.50	21.7	165.1	71.5

**Table 3:** Number of successive blows at first crack and at failure.

Mixture	Number of Blows at the First Crack (Nc)				Number of Blows at Failure (Nf)			
	28 Days	200 °C	400 °C	600 °C	28 Days	200 °C	400 °C	600 °C
PC	442	16	7	4	450	18	8	5
SFC 0.25%	2366	1056	386	104	2840	1312	544	146
SFC 0.5%	3678	1922	732	258	4244	2256	934	328
SFC 1%	6024	3748	1594	628	6860	4240	1904	720

## Data Availability

All data supporting the reported results can be found at (https://spectrum.library.concordia.ca/id/eprint/988373/) (accessed on January 2023).
